# Exploring the role of psychological flexibility in relationship functioning among couples coping with prostate cancer: a cross-sectional study

**DOI:** 10.1007/s00520-025-09229-8

**Published:** 2025-02-13

**Authors:** Hongen Ma, Laura Cariola, David Gillanders

**Affiliations:** Health in Social Science, University of Edinburgh, Edinburgh, EH8 9AG UK

**Keywords:** Prostate cancer, Couples, Psychological flexibility, Relationship satisfaction, Dyadic approach, Actor-partner interdependence model

## Abstract

**Objective:**

Prostate cancer (PCa) is an external stressor that can affect both patients and partners, but dyadic research in this area is limited. This study, guided by the vulnerability-stress-adaptation model, examines how PCa distress affects relationship satisfaction through psychological flexibility and self-esteem.

**Methods:**

The study used a cross-sectional design with a total sample size of 314 dyads. The actor-partner interdependence model was applied to examine both actor and partner effects. Covariates were also examined.

**Results:**

PCa distress negatively predicted psychological flexibility in both patients ($\beta$ = − .93, *p* < .001) and partners ($\beta$ = − 0.70, *p* < .001). Psychological flexibility, in turn, positively predicted self-esteem in both patients ($\beta$ = 0.19, *p* < .001) and partners ($\beta$ = 0.19, *p* < .001). Self-esteem significantly predicted relationship satisfaction for both patients ($\beta$ = 0.22, *p* < .001) and partners ($\beta$ = 0.22, *p* < .001). The indirect effects of PCa distress on relationship satisfaction via psychological flexibility and self-esteem were significant for both patients ($\beta$ = − .093, *p* < .001) and partners ($\beta$ = − .078, *p* < .001). Additionally, a significant indirect effect of partners’ PCa distress on patients’ relationship satisfaction was observed ($\beta$ = − .055, *p* < .01).

**Conclusion:**

The findings highlight the critical role of psychological flexibility and self-esteem in mediating the impact of PCa distress on relationship satisfaction for both patients and partners. Tailored interventions aimed at enhancing psychological flexibility to support relationship functioning in couples coping with PCa may be effective.

**Supplementary Information:**

The online version contains supplementary material available at 10.1007/s00520-025-09229-8.

## Introduction

In the UK, prostate cancer (PCa) is the most commonly diagnosed cancer in people assigned male at birth, with an incidence rate of approximately 52,300 cases per year [11]. Advances in early detection and treatment options, such as surgery, chemotherapy, and radiotherapy, have promisingly improved survival rates for PCa patients [90]. Yet, many survivors may still face long-term physical and psychosocial difficulties because of their diagnosis and treatments. The physical challenges associated with PCa, including irregular bowel movements, erectile dysfunction, and urinary incontinence, have been well documented in cancer research [69, 73]. Even so, the psychosocial impacts of PCa deserve more attention, especially the shared experiences among patients and their partners [114]. It is not uncommon for couples coping with cancer to undergo profound psychological distress, which can adversely affect their relationship dynamics and overall quality of life [110].

For patients, the experience of living with PCa may evoke feelings of vulnerability [106], fear of recurrence [94], changes in body image [29, 30], and concerns about masculinity [6], all of which can contribute to psychological distress. Partners, too, may often face psychological burdens, including caregiving stress [14], anxiety about the future [85], and changes in sexual intimacy [57]. This shared distress can strain couples’ relationships, affecting their communication, intimacy, and overall relationship satisfaction [19]. Intimate relationships play a vital role in supporting individuals through the cancer journey [84], yet the psychosocial impacts of cancer on relationship functioning remain under-researched [35]. A deeper understanding of how cancer-related distress affects the psychological and relational well-being of both patients and partners is critical to the development of effective interventions that can support couples in coping with the challenges of cancer.

Existing psychological interventions aimed at improving relationship functioning in couples coping with PCa have produced mixed results, often reflecting differences in their theoretical underpinnings. For example, an intervention [46] based on social cognitive theory did not improve relationship functioning for couples in the intervention group at 12-month follow-up. However, another intervention [51], rooted in the stress and coping framework [52], improved dyadic adjustment in the intervention group. The complexity of relationship dynamics, involving psychological, interpersonal, and contextual factors, can make it difficult to establish clear causal pathways [9]. However, without addressing the underlying mechanisms that might explain the impact of cancer-related distress on relationship outcomes, such interventions may have mixed or short-lived effects [5].

Despite the prevalence of psychological challenges faced by couples coping with PCa, less is known about the underlying mechanisms by which PCa-related distress may affect relationship outcomes. A more nuanced understanding of these mechanisms is essential for designing interventions that can sustainably improve relationship functioning in couples coping with PCa. In specific, who are the couples most at risk of having their relationship functioning affected by PCa? To answer this question, this present study drew on the vulnerability-stress-adaptation model (VSAM; [47]), which provides a comprehensive framework for describing and understanding relationship functioning under stress. Before the development of the VSAM, theories such as social exchange [56, 100], behaviour [34, 61], attachment [2, 7, 95], and crisis [67] provided valuable insights into the dynamics of intimate relationships. However, these theories were limited in their ability to fully account for the complexity of human relationships when considered independently. As a possible solution to this shortcoming, Karney and Bradbury [47] integrated these theories into the VSAM to capture a greater variety of the potential predictors and mechanisms of change in intimate relationships. In particular, the VSAM posits enduring vulnerabilities, stressful events, and adaptive processes as the three main domains that may interact in complex ways to influence relationship outcomes [21, 47]. Based on the VSAM framework, enduring vulnerabilities refer to long-term characteristics or dispositions of individuals, such as personality traits, cognitive tendencies, or emotional dispositions, which they bring to their relationships [44]. Stressful events are external circumstances or life challenges that can compromise an individual’s well-being and relationship functioning. Such stressful events are assigned to arise from a variety of sources, including illness, financial difficulties, or work-related stress [44]. Adaptive processes refer to the behaviours and strategies that individuals use to manage and respond to stressful events, as well as how they interact with others daily. As in the field of cancer research, different types of cancer have been identified as external life stressors that can lead to significant psychological distress among individuals [65]. Some diseases, including PCa, testicular cancer, and cervical cancer, have been shown to have negative impacts on patients’ sexual functions, which may further disrupt their intimate relationships [26, 92]. In the present study, we targeted PCa as an external life stressor and aimed to investigate the spill-over impact of PCa-related distress on couples’ relationship satisfaction.

According to the VSAM, external stressors can trigger adaptive processes. One such adaptive process supported by research is psychological flexibility, which may explain how couples cope with PCa distress [64, 103]. Psychological flexibility refers to a set of skills that enable individuals to act effectively in the presence of challenging thoughts, feelings, and emotions [39]. It included six key components: present moment awareness, values, committed action, self-as-context, defusion, and acceptance [21, 39]. Present moment awareness involves noticing where attention is focused and redirecting it to the present experience. Values clarify what is personally meaningful and guide behaviour in a purposeful way. Committed action refers to overt behavioural steps that are aligned with these values, particularly when such actions are taken even in the presence of internal or external barriers. Self-as-context allows for flexible perspective-taking, enabling individuals to observe their thoughts and feelings without being overly influenced by them. Cognitive defusion helps individuals to see their thoughts as mere thoughts rather than absolute truths. Finally, acceptance involves making room for unwanted experiences (e.g. thoughts, emotions, and sensations) without resisting them. In the context of this study, psychological flexibility may play a key role in mitigating the negative effects of PCa on relationship satisfaction. For example, if both patients and partners experience PCa as stressful, increasing their psychological flexibility may help them cope with these challenges and thereby improve their relationship satisfaction. Research has linked increased psychological flexibility to several cancer-related outcomes, such as improved emotional regulation [33], reduced cancer-related pain [25], and reduced fear of recurrence [94]. Moreover, the correlates of psychological flexibility in intimate relationships and family dynamics have been reviewed [21], and a previous study has demonstrated the positive effects of psychological flexibility on relationship quality and individual well-being [108].

As for the domain of enduring personal characteristics, self-esteem has been a commonly theorised and studied mechanism in cancer research [6, 70]. Self-esteem reflects an individual’s sense of self-worth and self-confidence, which tends to be stable over time, although it can fluctuate due to life events [83, 87]. In the context of this study, low self-esteem may serve as an enduring vulnerability that individuals bring into their relationships. Low self-esteem may predispose individuals to negative emotional reactions and difficulties in coping with stressful events, such as a cancer diagnosis or treatment side effects [76]. In addition, the experience of PCa can introduce multiple stressors, such as sexual dysfunction, body image issues, and caregiving burdens, which may exacerbate couples’ existing low self-esteem [3, 97]. For example, a patient’s treatment-related side effects may diminish their sense of masculinity and self-worth, which in turn may reduce their self-esteem. In addition, a partner’s ongoing caregiving responsibilities and witnessing the patient’s physical and emotional suffering may lead to feelings of helplessness or inadequacy in their supportive role and escalate their vulnerability to low self-esteem. As a result, couples may engage in maladaptive responses such as emotional withdrawal or reduced communication and intimacy [84]. A previous study showed that self-esteem was positively correlated with relationship quality in both breast cancer survivors and their spouses [55]. Another study also found positive relationships between self-esteem and relationship satisfaction in patients with breast cancer [20]. Finally, the predictive effects of psychological flexibility and masculine self-esteem on psychological distress and quality of life have also been discovered [66].

In summary, while PCa distress, psychological flexibility, self-esteem, and relationship satisfaction have been studied individually, their dynamic interaction within couples coping with PCa remains underexplored. Notably, no study has yet examined whether psychological flexibility mediates the relationship between cancer-related distress and relationship satisfaction at the dyadic level. From a clinical perspective, healthcare teams often struggle to provide tailored psychosocial support that can effectively meet the needs of both patients and caregivers due to the complexity of their experiences and diverse individual needs [96]. In addition, the limited number of studies investigating psychological support for couples coping with PCa has led to a lack of conclusive evidence on the most effective approaches, as well as a limited understanding of potential psychological factors that may influence relationship functioning [59]. Therefore, it should be explored whether mechanisms such as psychological flexibility and self-esteem can inform the design of psychosocial interventions to improve relationship satisfaction for both patients and their partners in supportive cancer care. To address this gap, we developed our conceptual framework grounded in the VSAM and existing evidence. We propose that higher PCa distress may lead to lower psychological flexibility in both patients and their partners. Lower psychological flexibility may contribute to lower self-esteem, which in turn may reduce overall relationship satisfaction. The selection of psychological flexibility and self-esteem directly aligns with our goal of understanding the relational dynamics underlying the impact of cancer-related distress on relationship satisfaction. These variables uniquely represent the adaptive processes and enduring characteristics that are essential for couples coping with chronic illness. While other factors (e.g. personality traits) offer valuable insights, psychological flexibility and self-esteem specifically address the dynamic adaptability and stable self-perception central to our VSAM-based approach. These factors are manipulable and can be targeted in psychological interventions in a way that other factors, such as personality, cannot.

To explore these interactions on the dyadic level, the actor-partner interdependence model (APIM; [49]) was used for the dyadic analysis. The APIM is a widely used tool for analysing dyadic data in the social sciences (e.g. [60, 89, 111]). The advantage of the model can be attributed to its ability to model the interdependence between dyad members, thereby facilitating the examination of both actor and partner effects. For example, the impact of a patient’s or a partner’s PCa distress on their own relationship satisfaction is referred to as an actor effect. In contrast, the impact of a patient’s or a partner’s PCa distress on their partner’s relationship satisfaction is referred to as a partner effect.

Based on the framework discussed above, it was hypothesised that (H1) PCa distress would negatively predict psychological flexibility, (H2) psychological flexibility would positively predict self-esteem, (H3) self-esteem would positively predict relationship satisfaction, and (H4) the indirect effect of PCa distress on relationship satisfaction through psychological flexibility and self-esteem would be significant. It was also expected that both actor and partner effects would be present, such that patients’ or partners’ PCa distress would negatively predict not only their own psychological flexibility but also their partners’ psychological flexibility.

## Methods

### Participants

This study aimed to recruit couples affected by PCa. This study aimed to recruit couples affected by PCa. The inclusion criteria were as follows: (1) couples where at least one partner had a confirmed diagnosis of PCa, including those in early stages, with precancerous cells, or who had undergone any form of treatment, (2) both partners were English speaking and either resided in or had received healthcare in the UK, (3) both partners were aged 18 or older, (4) couples were in a monogamous relationship or marriage for at least six months, and (5) both partners were capable of providing informed consent and completing the study. Couples were excluded from the study if either partner had cognitive impairments (e.g. head injury) or a terminal comorbid condition (e.g. dementia) that could affect their ability to provide informed consent and complete the questionnaires.

### Procedure

This study received approval from the University of Edinburgh’s Social Science Research Ethics Committee (22-23CLPS152) before participant recruitment. The research team collaborated with the charity Prostate Scotland to distribute study posters to potential participants. To increase the size and heterogeneity of the sample, paid Facebook advertisements and social media posts (on platforms such as X and Reddit) were also used. A hyperlink embedded in these posts directed participants to the study website hosted on Qualtrics, where they read the participant information sheets regarding their ethical rights following the British Psychological Society (BPS) guide on research with human participants, provided their online consent, and completed the survey between January and August 2024 [77].

To obtain dyadic data, personal information such as name and postcode was used to match responses. For ethical reasons, all personal information was removed after data matching. A total of 315 couples were identified, including one same-sex couple. Before applying the APIM, it is necessary to categorise dyads as either distinguishable or indistinguishable. For example, dyads such as same-sex couples or identical twins are theoretically indistinguishable because there is no meaningful variable that distinguishes the two members. However, heterosexual couples or parent–child pairs are distinguishable because there is a meaningful variable, such as gender or role, which consistently differentiates the members of all dyads [54]. Given the small number of same-sex couples, it was impractical to run another model with indistinguishable data. Therefore, data from 314 heterosexual couples were included in the final APIM analysis. As for the same-sex couple, their data were presented separately in a descriptive manner to ensure inclusivity while maintaining methodological integrity. The de-identified data were stored in a password-protected research data repository at the university following relevant data protection regulations.

### Measures

#### Demographics

A demographic questionnaire was used to collect information about participants, including age, ethnicity, country of residence in the UK, length of relationship, marital status, education level, employment status, year of PCa diagnosis, stage of PCa, and other relevant characteristics.

#### Prostate cancer distress

The Impact of Events Scale-Revised (IES-R; [112]) was used to measure PCa distress. It consists of 22 items that can assess 3 facets of distress related to coping with stressful life events, such as cancer, experienced by participants in the past 7 days, including intrusion (e.g. ‘Any reminder brought back feelings about it’), avoidance (e.g. ‘I tried not to talk about it’), and hyperarousal (e.g. ‘I felt irritable and angry’). Participants rated each item on a 5-point scale from 0 (not at all) to 4 (extremely). A high total score (range 0–88) indicates severe psychological distress. The IES-R has been shown to have adequate psychometric properties in the context of cancer research [43, 81, 107]. In this study, the internal consistency of the IES-R was excellent for patients (Cronbach’s α = 0.94) and partners (Cronbach’s α = 0.95).

#### Psychological flexibility

Psychological flexibility was assessed using the Comprehensive Assessment of Acceptance and Commitment Therapy Processes (CompACT; [28]). The CompACT targets three constituent elements of psychological flexibility: openness to experience (e.g. ‘Thoughts are just thoughts, and they don’t control what I do’), behavioural awareness (e.g. ‘I find it difficult to stay focused on what’s happening in the present’), and valued action (e.g. ‘I act in ways that are consistent with how I wish to live my life’). It comprises 23 items, each rated on a 7-point scale, with 0 indicating a strong disagreement and 6 indicating a strong agreement. The total score ranges from 0 to 138, with a higher score indicating greater psychological flexibility. The scale has been used in cancer studies and has good internal consistency ratings [66, 94]. In the present sample, the internal consistency was adequate for patients (Cronbach’s $\alpha$ = 0.91) and partners (Cronbach’s $\alpha$ = 0.90).

#### Self-esteem

The Rosenberg Self-Esteem Scale (RSES; [86] was used to measure general levels of self-esteem. Unlike the Masculine Self-Esteem Scale (MSES; [17]), the RSE provides a broad assessment of an individual’s overall sense of worth and importance, making it applicable to both PCa patients and their partners in this study (e.g. ‘I take a positive attitude toward myself’). It is a 10-item scale with a total score range from 0 to 30. A higher score over 25 indicates higher self-esteem. The RSE has been validated as a reliable measure among cancer studies [4, 8, 16]. In this study, the internal consistency of RSES was strong for both patients (Cronbach’s α = 0.93) and partners (Cronbach’s α = 0.93).

#### Relationship functioning

The Relationship Assessment Scale (RAS; [40]) was employed to evaluate relationship satisfaction among patients and their partners. The scale comprises seven items (e.g. ‘How good is your relationship compared to most’). The items are rated on a 5-point scale, ranging from 1 (indicating low satisfaction) to 5 (indicating high satisfaction). Total scores can range from 7 to 35, with higher scores indicating greater relationship satisfaction. Previous research in the field of oncology has reported sufficient psychometric properties of the RAS [10, 109]. In the present study, the RAS’s internal consistency was good for both patients (Cronbach’s $\alpha$ = 0.87) and partners (Cronbach’s $\alpha$ = 0.85).

### Data analysis

The descriptive characteristics of the data were first evaluated. The percentage of missing data for each numeric variable was calculated, and Little’s missing completely at random (MCAR) test was conducted. The results indicated that an average of 0.42% of data (range: 0.04–2.87%) was missing across variables and likely to be MCAR (${c}^{2}$ = 4548, *p* = 0.218). As a result, multiple imputation (MI) using predictive mean matching (PMM) was employed to replace missing data at the individual item level. MI-PMM is particularly well-suited for continuous variables, as it avoids imputing values outside the observed data range, thus preserving the integrity of the distributions [62].

Outliers were identified through visual inspection of boxplots and analysis of z-scores. A few data points with z-scores greater than three were identified as potential outliers [24]; however, they were not removed as they fell within the expected range of the measures and did not indicate extreme deviations. This approach preserved natural variability without distorting the results [102]. Normality was assessed by visual inspection and the overall values between − 2 and 2 of skewness and kurtosis [104]. Although patients’ relationship satisfaction with a skewness of − 1.60 and a kurtosis of 2.34 showed minor deviations from normality, these were not severe enough to warrant concern. Given the large sample size (*N* = 314 dyads), Pearson correlation was used to assess bivariate correlations between patients’ and partners’ outcome measures. Paired samples *t*-tests were also conducted to evaluate mean differences between patients’ and partners’ outcome measures. Cohen’s *d* (i.e. standardised mean difference) was calculated to determine if a mean difference was large or small. Multicollinearity was assessed using the variance inflation factor (VIF), and Harman’s single-factor test was performed to check for common method bias. The VIF values were all below 5, indicating that there was no significant issue of multicollinearity [98]. Also, the Harman’s single-factor test revealed that the first factor explained 28.8% of the variance, suggesting that common method bias was unlikely to be a major concern [68].

Covariates were evaluated before conducting the primary analysis of actor and partner effects. Continuous demographic variables (e.g. age, relationship length) were assessed using Pearson correlation, while categorical variables (e.g. education, employment) were analysed with ANOVA. Only those demographic variables significantly associated with the dependent variables were included as covariates in the APIM to control for potential confounding effects.

**Fig. 1:**
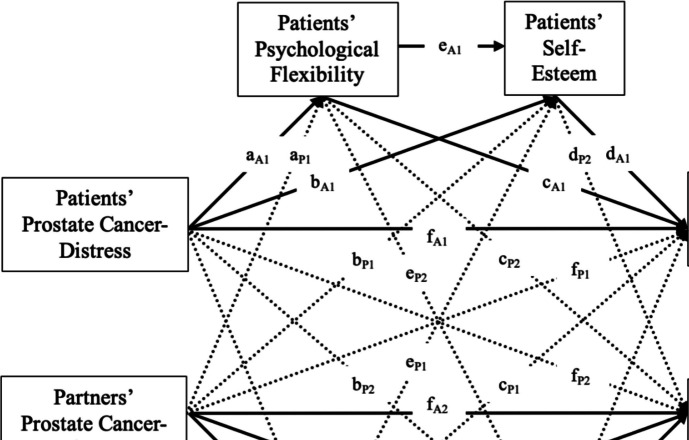
Conceptual actor-partner mediation model

The primary analysis utilised the APIMeM, an extended version of the APIM with mediation [54]. The APIMeM allowed us to explore how each partner’s PCa distress affects their own and their partner’s relationship satisfaction through the serial mediating roles of psychological flexibility and self-esteem (see Fig. 1). Both direct and indirect pathways were examined using structural equation modelling (SEM), which was preferred for distinguishable dyads over alternatives such as multilevel modelling [53]. The primary analysis followed a three-stage process. First, we estimated a full APIMeM, including both actor and partner effects and all direct and indirect paths. To assess interdependence, we conducted a chi-square difference test comparing the full APIMeM with an actor-only APIMeM. A significant improvement in model fit when partner effects were included indicated meaningful partner effects and the presence of interdependence. In addition, to determine whether the dyad members (patients and partners) were distinguishable regarding model parameters, we compared the full model with a constrained model, where all parameters were set equal across dyad members. The chi-square difference test assessed whether this constraint led to a significant reduction in model fit. A significant result indicated that dyad members were distinguishable, supporting the use of the full model. Second, we used Wald’s test to assess the significance of individual parameters, identifying whether any parameters could be statistically constrained to equality without a significant loss of model fit. Non-significant effects were flagged for potential restriction. Finally, based on Wald’s test results, we developed a more parsimonious model by constraining non-significant parameters. The simplified model retained the essential actor and partner effects while reducing the overall complexity of the model without compromising its overall fit.

Model fit was assessed using several indices: the root-mean-square error of approximation (RMSEA), the standardised root-mean-square residual (SRMR), and the comparative fit index (CFI). A good model fit was indicated by an RMSEA below 0.06, an SRMR under 0.08, and a CFI above 0.95 [41]. Due to the general assumption of nonnormal distribution of indirect effects, indirect effects were tested using bootstrapping with 5000 resamples [99]. In this study, a serial mediation effect was observed if the serial indirect effect was significantly different from zero. All the analyses were conducted in RStudio (2023.12.1 + 402) with packages such as lavaan [88]. The interpretation of the results was based on both the statistical significance (*p* < 0.05) and the bootstrapped 95% confidence intervals, which did not include zero for the indirect associations. Correlations of 0.15, 0.25, and 0.35 were considered to be small, medium, and large, respectively [31]. Finally, Cohen’s *d* of 0.2, 0.5, and 0.8 were considered small, medium, and large [50].

### Sample size

There is limited guidance on power analysis and sample size calculation for mediation models, particularly those involving the APIM with multiple mediators [93]. Therefore, a power analysis was conducted for the APIM, followed by a power analysis for the serial mediation model. Following previous studies, the power analysis for APIM was done through the APIMPowerR program [1]. The results indicated that a minimum of 197 dyads (197 patients and 197 partners) was required to achieve sufficient statistical power (0.95), assuming a medium effect size (0.25) and a significance level (α = 0.05). In contrast, a Monte Carlo simulation [72] was employed to perform a power analysis for the sequential mediation model, which indicated a minimum sample size of 300 individuals was necessary to achieve the sample power level (0.95), effect size (0.25), and significance level (α= 0.05). Given the complexity of APIMeM, we followed the more conservative recommendation from the serial mediation model, aiming to recruit a minimum sample size of 300 dyads (300 patients and 300 partners) to ensure robust statistical power.

## Results

### Descriptive analysis

The final sample’s demographic information can be seen in Table 1. The mean age of patients (*M* = 69, *SD* = 5.4) was higher compared to the mean age of partners (*M* = 65, *SD* = 5.7). The length of the relationship ranged from 11 to 55 years, with an average of 37 years. Most couples were married (95.5%) and resided in England (78%). Most participants were white (99.4% of patients and 99% of partners), attended some college (30.6% of patients and 27.8% of partners), were retired (73.6% of patients and 60.8% of partners), and had not sought any psychological support (86.9% of patients and 90.4 of partners). Among patients, most had early-stage PCa (61.9%) and had undergone surgery (34.7%), radiation therapy (48.4%), and hormone therapy (48.1%).

**Table 1:** Sample characteristics of patients and partners (*N* = 314 dyads)

Variable	Patient		Partner	
	*M*	*SD*	*M*	*SD*
Age (years)	69.0	5.4	65.3	5.7
Relationship length (years)*	36.6	10.5	36.4	10.5
Years since diagnosis	4.3	3.7		
	*N*	*%*	*N*	*%*
Gender				
Male	314	100.0	0	0.0
Female	0	0.0	314	100.0
Ethnicity				
White	309	99.4	307	99.0
Asian	2	0.7	1	0.3
Other	0	0.0	2	0.7
UK areas of residence				
England	245	78.0	245	78.0
Isle of Man	1	0.3	1	0.3
Northern Ireland	5	1.6	5	1.6
Scotland	43	13.7	43	13.7
Wales	20	6.4	20	6.4
Sexual orientation				
Heterosexual	314	100.0	314	100.0
Relationship status				
Married	300	95.5	300	95.5
Never married	14	4.5	14	4.5
Education				
2-year degree	11	3.5	13	4.2
4-year degree	27	8.7	21	6.7
Doctorate	5	1.6	9	2.9
High school	45	14.5	69	22.0
Less than high school	53	17.0	41	13.1
Professional degree	75	24.1	73	23.3
Some college	95	30.6	87	27.8
Employment				
Disabled	6	1.9	14	4.5
Employed full time	53	16.9	44	14.0
Employed part-time	18	5.7	56	17.8
Retired	231	73.6	191	60.8
Unemployed and looking for work	1	0.3	9	2.9
Unemployed and not looking for work	5	1.6	0	0.0
Psychological support				
No	273	86.9	284	90.4
Yes	41	13.1	30	9.6
Phase of prostate cancer				
Advanced	45	14.4		
Early stage	193	61.9		
Locally advanced	74	23.7		
Treatment type*				
Active surveillance/monitoring	28	8.9		
Brachytherapy	9	2.9		
Chemotherapy	32	10.2		
Considering options	1	0.3		
Hormone therapy	151	48.1		
None	4	1.3		
Other	7	2.2		
Radiation therapy (external beam)	152	48.4		
Surgery	109	34.7		

^*^Reported relationship lengths varied among several couple members. Multiple treatments were reported

Based on the paired samples *t*-tests (see Table 2), the mean differences in PCa distress (*t* = − 3.73, *b* = − 0.2, *p* < 0.001), psychological flexibility (*t* = 4.82, *b* = 0.3, *p* < 0.001), and self-esteem (*t* = 3.28, *b* = 0.2, *p* < 0.01) were small but significant between patients and partners. There were no significant mean differences in the relationship satisfaction scores between patients and partners (*t* = 1.69, *p* = 0.091). Furthermore, all variables were correlated in the expected directions, as previously stated in the hypotheses (see Table 3). PCa distress was negatively correlated with psychological flexibility for both patients (*r* = − 0.69, *p* < 0.001) and partners (*r* = − 0.63, *p* < 0.001). Psychological flexibility was positively correlated with self-esteem (patients: *r* = 0.77, *p* < 0.001; partners: *r* = 0.72, *p* < 0.001). Finally, self-esteem was positively correlated with relationship satisfaction (patients: *r* = 0.42, *p* < 0.001; partners: *r* = 0.30, *p* < 0.001). The assessment of demographic variables did not identify any variables that met the criteria for inclusion as covariates.

**Table 2:** Means, standard deviations, and reliabilities of outcome variables

Variable	Patients			Partners			Paired samples *t*	Cohens’ *d*
	*M*	*SD*	Cronbach’s *α*	*M*	*SD*	Cronbach’s *α*		
PCa distress	26.32	17.14	0.94	31.80	19.57	0.95	** − 3.73**	** − 0.21**
Self-esteem	30.71	6.57	0.93	29.07	5.93	0.93	**3.28**	**0.19**
Psychological flexibility	88.66	23.14	0.91	80.05	21.55	0.90	**4.82**	**0.27**
Relationship satisfaction	31.03	4.86	0.87	30.37	4.89	0.85	1.69	0.12

*PCa* prostate cancer. Note: Significant results are in bold (*p* < 0.05)

**Table 3:** Correlations matrix of study variables

Variables	1	2	3	4	5	6	7	8
1. Relationship satisfaction_Patient								
2. PCa distress_Patient	** − 0.31**							
3. Self-esteem_Patient	**0.42**	** − 0.59**						
4. Psychological flexibility_Patient	**0.36**	** − 0.69**	**0.77**					
5. Relationship satisfaction_Partner	**0.38**	** − 0.13**	**0.22**	**0.13**				
6. PCa distress_Partner	** − **0.08	0.02	** − **0.06	0.02	** − 0.16**			
7. Self-esteem_Partner	**0.12**	** − **0.01	0.03	0.00	**0.30**	** − 0.50**		
8. Psychological flexibility_Partner	**0.14**	** − **0.02	0.03	0.01	**0.25**	** − 0.63**	**0.72**	

*PCa* prostate cancer. Note: Significant results are in bold (*p* < 0.05)

In the same-sex couple, both the patient and the partner were 60 years old, never married, together for 17 years, and living in England. The patient had been diagnosed with locally advanced cancer and had received radiation and hormone therapies. Both the patient and the partner reported similar levels of relationship satisfaction (patient: 33; partner: 34). However, the patient reported lower PCa distress (patient: 6; partner: 18), higher psychological flexibility (patient: 123; partner: 76), and higher self-esteem (patient: 40; partner: 26) compared to the partner.

### APIMeM

**Fig. 2:**
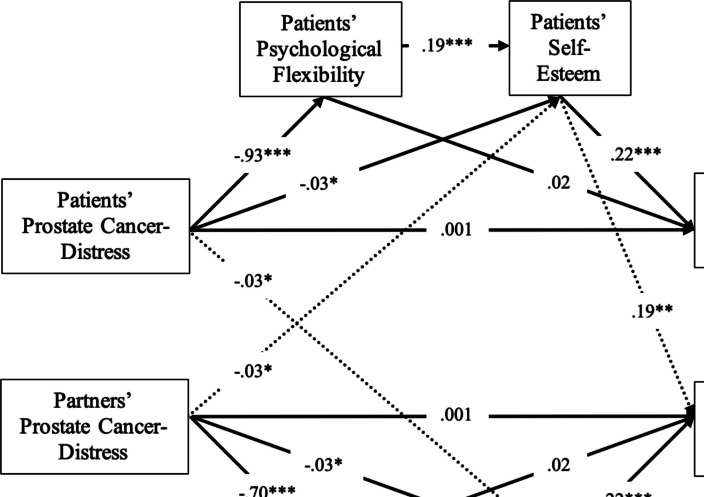
Model path results (*p < .05, **p < .01, ***p < .001)

The full APIMeM was a completely saturated model (*df* = 0, *CFI* = 1.000, *RMSEA* = 0.000, *SRMR* = 0.000). The chi-square difference test revealed that actor-only APIMeM (${c}^{2}$ = 32.683, *df* = 12, *p* < 0.01, *CFI* = 0.979, *RMSEA* = 0.074, *SRMR* = 0.056) reduced the model fit, suggesting evidence of interdependence between patients and partners. The chi-square difference test also indicated the patients and partners were statistically distinguishable, as the constrained APIMeM showed a worse model fit (${c}^{2}$ = 24.058, *df* = 12, *p* = 0.020, *CFI* = 0.988, *RMSEA* = 0.057, *SRMR* = 0.069). Based on the results of Wald’s test, the parsimonious APIMeM was built with only non-significant parameters constrained. The non-significant chi-square difference test supported the use of the parsimonious APIMeM (${c}^{2}$ = 10.956, *df* = 9, *p* = 0.279, *CFI* = 0.998, *RMSEA* = 0.0026, *SRMR* = 0.034) over the full APIMeM. Since the parsimonious APIMeM did not compromise the model fit, it was preferable for interpretation and reporting due to its simplicity and efficiency. The diagram of the APIMeM was displayed (see Fig. 1), and the results of direct and indirect effects were included (see Fig. 2 and Table 4).

**Table 4:** Direct and indirect effect results

Effect	Estimate	95% *CI*	*p*	Proportion (%)
Patient’s actor effect				
Total effect	** − .092**	**[− 0.126, − .060]**	** < .001**	
Total indirect effect	** − .093**	**[− 0.136, − .055]**	** < .001**	**99**
PCD_T → PF_T → SE_T → RS_T	** − .066**	**[− .090, − .046]**	** < .001**	**71**
PCD_T → PF_T → SE_R → RS_T	** − **.021	[**− **.044, − .000]	.052	23
PCD_T → PF_R → SE_R → RS_T	** − **.000	[**− **.004, .004]	0.959	00
PCD_T → PF_R → SE_T → RS_T	** − **.005	[**− **.012, .001]	0.102	05
Direct effect: PCD_T → RS_T	.001	[**− **.025, .027]	0.940	01
Partner’s actor effect				
Total effect	** − .077**	**[− 0.104, − .050]**	** < .001**	
Total indirect effect	** − .078**	**[− 0.111, − .048]**	** < .001**	**99**
PCD_R → PF_R → SE_R → RS_R	** − .052**	**[− .068, − .036]**	** < .001**	**66**
PCD_R → PF_R → SE_T → RS_R	** − .016**	**[− .033, − .000]**	**.047**	**20**
PCD_R → PF_T → SE_T → RS_R	** − **.004	[**− **.012, .001]	0.185	05
PCD_R → PF_T → SE_R → RS_R	** − **.006	[**− **.014, − .000]	.062	08
Direct effect: PCD_R → RS_R	.001	[**− **.025, .027]	0.940	01
Patient’s partner effect				
Total effect	** − **.021	[**− **.068, .026]	0.373	
Total indirect effect	** − **.028	[**− **.077, .025]	0.281	88
PCD_T → PF_R → SE_R → RS_R	** − **.004	[**− **.012, .003]	0.266	08
PCD_T → PF_R → SE_T → RS_R	** − **.005	[**− **.013, .001]	0.162	10
PCD_T → PF_T → SE_T → RS_R	** − .027**	**[− .052, − .003]**	**.028**	**53**
PCD_T → PF_T → SE_R → RS_R	.009	[**− **.022, .043]	0.603	17
Direct effect: PCD_T → RS_R	.006	[**− **.019, .032]	0.633	12
Partner’s partner effect				
Total effect	** − .049**	**[− .084, − .015]**	**.005**	
Total indirect effect	** − .055**	**[− .096, − .018]**	**.005**	**90**
PCD_R → PF_T → SE_T → RS_T	** − **.004	[**− **.012, .003]	0.266	07
PCD_R → PF_T → SE_R → RS_T	** − **.000	[**− **.004, .004]	0.820	00
PCD_R → PF_R → SE_R → RS_T	** − .026**	**[− .045, − .008]**	**.006**	**43**
PCD_R → PF_R → SE_T → RS_T	** − .024**	**[− .048, − .001]**	**.041**	**40**
Direct effect: PCD_R → RS_T	.006	[**− **.019, .032]	0.633	10

*PCD* prostate cancer distress, *PF* psychological flexibility, *SE* self-esteem, *R* partner, *T* patient. Note: Significant results are in bold (*p* < 0.05)

For patients’ actor effects, PCa distress had a significant negative effect on psychological flexibility ($\beta$ = − 0.93, *p* < 0.001), suggesting that higher levels of distress were associated with lower levels of psychological flexibility. PCa distress also had a significant negative effect on self-esteem ($\beta$ = − 0.03, *p* = 0.036), although this effect was smaller. Additionally, psychological flexibility positively predicted self-esteem ($\beta$ = 0.19, *p* < 0.001), indicating that patients with greater psychological flexibility also had higher self-esteem. However, PCa distress did not have a significant direct effect on relationship satisfaction ($\beta$ = 0.001, *p* = 0.940). Self-esteem, on the other hand, was positively associated with relationship satisfaction ($\beta$ = 0.22, *p* < 0.001), while psychological flexibility had a marginally non-significant effect on relationship satisfaction ($\beta$ = 0.02, *p* = 0.053). For partners’ actor effects, a similar pattern was observed. Partners’ PCa distress had a significant negative effect on their psychological flexibility ($\beta$ = − 0.70, *p* < 0.001), indicating that higher distress was linked to lower psychological flexibility. Partners’ PCa distress also significantly predicted lower self-esteem ($\beta$ = − 0.03, *p* = 0.036), and psychological flexibility positively predicted self-esteem ($\beta$ = 0.19, *p* < 0.001). Partners’ PCa distress did not have a significant direct effect on relationship satisfaction ($\beta$ = 0.001, *p* = 0.940), but self-esteem was positively associated with relationship satisfaction ($\beta$ = 0.22, *p* < 0.001). Psychological flexibility had a marginally non-significant effect on relationship satisfaction in partners ($\beta$ = 0.02, *p* = 0.053). Regarding the partner effects, patients’ self-esteem positively predicted partners’ relationship satisfaction ($\beta$ = 0.19, *p* < 0.01). In addition, patients’ PCa distress was negatively correlated with partners’ self-esteem ($\beta$ = − 0.03, *p* = 0.046) and vice versa for partners ($\beta$ = − 0.03, *p* = 0.046).

#### Direct effects

The actor and partner effects for both patients and partners were examined to understand the impact of PCa distress on relationship satisfaction. The direct effect of patients’ PCa distress on their relationship satisfaction was non-significant ($\beta$ = 0.001, *p* = 0.940), accounting for only 0.01% of the total effect. Similarly, the direct effect of partners’ PCa distress on their relationship satisfaction was also non-significant ($\beta$ = 0.001, *p* = 0.940), representing 0.01% of the total effect. Examining the partner effects, the direct effect of patients’ PCa distress on their partners’ relationship satisfaction was non-significant ($\beta$ = 0.006, *p* = 0.633), accounting for 12% of the total effect. Likewise, the direct effect of partners’ PCa distress on patients’ relationship satisfaction was non-significant ($\beta$ = 0.006, *p* = 0.633), representing 10% of the total effect.

#### Indirect effects

Despite the non-significant direct effects, significant indirect serial mediation pathways were observed. For patients, the total indirect effect of PCa distress on their relationship satisfaction was significant ($\beta$ = − 0.093, *p* < 0.001), accounting for 99% of the total effect. The strongest pathway contributing to this indirect effect was through psychological flexibility (patients) and self-esteem (patients), with an indirect effect of − 0.066 (*p* < 0.001), which represented 71% of the total effect. For partners, the total indirect effect of PCa distress on their relationship satisfaction was significant ($\beta$ = − 0.077, *p* < 0.001), representing 99% of the total effect. The primary indirect pathway involved psychological flexibility (partners) and self-esteem (partners), which contributed an indirect effect of − 0.052 (*p* < 0.001), making up 66% of the total effect. Another significant pathway from PCa distress (partners) to relationship satisfaction (partners) via psychological flexibility (partners) and self-esteem (patients) had an effect of − 0.016 (*p* = 0.047), accounting for 20% of the total effect.

Additionally, partner effects revealed significant indirect effects of partners’ PCa distress on patients’ relationship satisfaction, with a total indirect effect estimate of − 0.049 (*p* < 0.01), accounting for 90% of the total effect. The pathway involving psychological flexibility (partners) and self-esteem (partners) contributed 43% of the total effect (*p* < 0.01), while the pathway involving psychological flexibility (partners) and self-esteem (patients) had a significant contribution of 40% (*p* = 0.041). For patients, the indirect effects of their PCa distress on their partners’ relationship satisfaction were largely non-significant. However, one significant pathway emerged through psychological flexibility (patients) and self-esteem (patients), with an indirect effect of − 0.027 (*p* = 0.028), accounting for 53% of the total effect.

## Discussion

Understanding the impact of PCa distress on relationship functioning is critical, especially given the increasing number of patients diagnosed with PCa and the growing caregiving role assumed by patients’ partners. Physical and psychosocial challenges associated with PCa, such as treatment side effects and emotional distress, are likely to reduce the time and energy that couples can devote to maintaining a satisfying relationship during the cancer journey [19]. Despite these concerns, there is limited research on the mechanisms that link cancer-related distress to relationship satisfaction. To identify which couples might be at greater risk of relationship deterioration during this journey, we drew upon the vulnerability-stress adaptation model (VSAM; [47]) and existing evidence. The VSAM highlights the substantial impact of the interplay between personal vulnerabilities, life stressors, and adaptive processes in shaping the trajectory of relationship well-being throughout the cancer experience. To the best of our knowledge, this study is the first to examine the mediating roles of psychological flexibility and self-esteem in the relationship between PCa distress and relationship satisfaction, considering both individual and dyadic effects. The results yield several notable findings, which are discussed in detail in the following sections.

One of the key findings of this study is the remarkable similarity between patients and partners in the psychological pathways examined. As predicted by our first hypothesis (H1) regarding actor effects, PCa distress negatively predicted psychological flexibility in both patients and partners. This negative association suggests that higher levels of PCa distress may hinder individuals’ ability to remain adaptable when faced with significant emotional and psychological challenges. This finding is consistent with previous research showing that chronic illness can negatively impact emotional regulation and psychological adaptability [4]. Consistent with our second hypothesis (H2) for actor effects, psychological flexibility was found to positively predict self-esteem for both patients and partners. As shown in previous research [15, 63], individuals with greater psychological flexibility may tend to maintain a more positive self-concept, which contributes to higher self-esteem. This relationship was consistent for both patients and partners, suggesting that greater psychological flexibility may help to maintain self-esteem, even in the presence of external stressors such as PCa. In addition, self-esteem was positively associated with relationship satisfaction in both patients and partners, as hypothesised for actor effects (H3). Consistent with previous research [37], this suggests that individuals with higher self-esteem are more likely to experience satisfaction in their romantic relationships, reinforcing the idea that an individual’s self-perception may play a critical role in their relationship experience. Taken together, these findings support our final hypothesis (H4) regarding actor effects, as PCa distress indirectly affected relationship satisfaction through the serial mediation of psychological flexibility and self-esteem in both patients and partners. Importantly, the direct effects of PCa distress on relationship satisfaction were not significant for either group. This suggests that PCa distress may not directly lead to lower relationship satisfaction but may exert its influence indirectly through psychological flexibility and self-esteem.

These findings support the VSAM, which explains how cancer-related distress affects relationship satisfaction by demonstrating the interaction between adaptive processes and enduring characteristics. Specifically, we observed that PCa distress had a stronger association with psychological flexibility than with self-esteem or relationship satisfaction. This suggests that adaptive processes, such as psychological flexibility, may be more responsive to the present psychological impact of PCa distress, likely due to their role in coping with both external and internal stressors. PCa distress, as a stressor, may fluctuate based on factors such as treatment stage, symptom severity, and emotional reactions [78]. Psychological flexibility, as an adaptive process, similarly adapts to these stressors, making it a key factor in coping with chronic illness [45]. This dynamic responsiveness may explain why PCa distress had a strong predictive effect on psychological flexibility in both patients and partners. However, the smaller, non-significant predictive effect of PCa distress on relationship satisfaction may be understood through the lens of trait stability. Relationship satisfaction is relatively more stable and enduring, remaining constant in the absence of major upheaval [48]. This stability may explain the weaker influence of situational variables such as PCa distress and psychological flexibility on relationship satisfaction. Self-esteem, as an enduring trait, may also be relatively stable in the presence of short-term fluctuations caused by external stressors. As demonstrated by previous research [80], self-esteem may undergo systematic changes over the course of an individual’s lifetime yet remain relatively stable at different stages of development. Therefore, the significant correlation between self-esteem and relationship satisfaction observed in our study may be due to the fact that both variables exhibit a comparable degree of stability, making them less susceptible to the more dynamic aspects of PCa distress. Overall, given the cross-sectional nature of this study, we cannot infer causality or temporal sequencing; however, the different strength of associations observed here underscores the importance of examining both adaptive and stable traits when exploring how couples cope with cancer-related distress. This nuanced view may guide interventions that prioritise fluid behavioural mechanisms, such as psychological flexibility, to support relationship satisfaction during the cancer journey.

While similarities in psychological pathways were observed between patients and partners, some important differences also emerged. There was a significant correlation in relationship satisfaction between patients and partners; however, no significant intercouple correlations were found for other variables, such as psychological flexibility and self-esteem. This lack of partner effects supporting H1, H2, and H3 suggests that patients and partners may represent two distinct groups, although they may follow similar pathways. The distinction between patients and partners was confirmed by the results of the actor-partner interdependence model (APIM; [49]), which demonstrated the statistical significance of distinguishability. Two main factors may explain the existence of distinguishability. First, patients have a direct, personal experience with PCa as those diagnosed with the disease. This direct exposure to the physical and emotional challenges of cancer can create a unique psychological burden for patients [82], which may explain why their psychological outcomes can differ from their partners’ outcomes. Second, the gender difference between patients and partners may also lead to distinguishability. As aforementioned, the heterosexual couples included in our study may theoretically be distinguishable dyads because of the gender factor. Gender differences in social science research have been widely documented, with male and female partners often exhibiting different coping styles and psychological outcomes [27, 58, 101].

In addition, our study found significant mean differences in PCa distress, psychological flexibility, and self-esteem between patients and partners. Although these differences were small, they were clinically relevant when considering commonly observed gender differences in psychological outcomes. These patterns may reflect the emotional burden of caregiving, as partners often report feeling emotionally overwhelmed in dealing with the emotional, logistical, and sometimes financial demands of the disease [18]. This is consistent with previous research indicating that partners of cancer patients may experience higher levels of psychological distress than the patients themselves [32, 71]. Partners’ lower self-esteem may be related to feelings of helplessness in alleviating the patient’s suffering, which may reduce their sense of personal efficacy. Previous research similarly highlights that caregivers often experience lower self-esteem when they perceive their caregiving efforts as inadequate [42, 74]. In addition, partners may not receive the same level of social or medical support as patients, which may further contribute to their psychological distress and reduced flexibility [38]. Overall, male patients may often experience distress related to their identity and physical well-being, while female partners may assume a caregiving role, which may result in discrepancies in psychological flexibility and self-esteem. These findings point to the unique psychological experiences of patients and partners in coping with PCa.

Finally, the finding of a few significant indirect partner effects supporting H4 provides deeper insights into the interdependence between patients and partners in coping with PCa. It is common in dyadic research to discover stronger actor effects than partner effects [12]. However, the presence of some significant partner effects suggests a degree of interdependence between these two groups, even though patients and partners may function as distinct entities. The results of the APIM provided evidence of this interdependence, in particular by showing that partners had a more significant partner effect than patients. This suggests that partners’ distress may have a stronger influence on patients’ relationship satisfaction through the mediation of psychological flexibility and self-esteem than vice versa. As noted above, partners may serve as patients’ primary caregivers, devoting significant time and resources to managing and supporting patients’ needs. This caregiving role can create a dynamic in which partners’ distress can have a disproportionate impact on the patient, as patients become increasingly dependent on their partners for emotional and physical support (Q. [14, 97]). This finding underscores the concept of interdependence, whereby one partner’s distress can spill over and affect the other’s relationship satisfaction, revealing the dyadic nature of coping with cancer [13, 36].

### Limitations and future research

Although this study provides valuable insights into the psychological mechanisms underlying the association between PCa distress and relationship satisfaction, several limitations should be acknowledged. First, the cross-sectional design of the study limits the ability to draw definitive conclusions regarding causality. Although we hypothesised certain pathways based on theoretical frameworks such as the VSAM and existing evidence, the directionality of these relationships cannot be determined with certainty. Thus, future longitudinal studies that follow couples over time would provide more robust evidence for the temporal ordering of variables and clarify whether changes in psychological flexibility and self-esteem precede changes in relationship satisfaction. Second, the use of self-report measures may introduce bias, as participants’ responses may be influenced by social desirability or recall inaccuracies. This is particularly relevant in studies of sensitive topics such as cancer-related distress and relationship satisfaction. Although we used validated instruments to measure the constructs, future research should consider incorporating more objective measures, such as behavioural assessments or physiological stress markers, to complement self-report measures. In addition, the inclusion of third-party observations (e.g. clinicians and other family members) may also help to alleviate concerns regarding potential biases inherent in self-report data.

Third, the couples included may not fully capture the diversity of experiences among those coping with PCa, particularly in terms of gender, cultural, socioeconomic, and geographic variability. Despite our efforts to recruit a large representative sample via a variety of methods (e.g. social media, cancer charities, and community posters), the majority of participants were from the UK, predominantly white, and had higher levels of education, which may limit the generalizability of the findings to other populations. In addition, the exploration of relationship dynamics in non-heterosexual relationships was limited due to the lack of matched data from non-heterosexual couples. The dynamics of coping with chronic illness, relationship satisfaction, and psychological processes may differ across different relationship types due to unique stressors, societal pressures, and relational experiences that affect same-sex couples [22, 105]. Future research should aim to recruit more diverse samples representing a range of cultural, gender, and socioeconomic backgrounds, as well as non-heterosexual couples. This would not only increase the generalizability of findings but also contribute to the development of more inclusive and tailored interventions that address the unique needs of diverse populations.

Finally, there may be other unmeasured variables that could also serve as potential mechanisms. For example, attachment style and broader personality traits, such as those in the Big Five model, have also been found to be associated with relationship satisfaction [12, 79]. However, our selection of psychological flexibility and self-esteem was driven by their alignment with the VSAM, and the targeted focus allowed for a clearer analysis of how couples cope with the psychological impact of cancer-related distress. Including a broader range of variables, while potentially enriching, would shift the focus of the study and require a broader theoretical and methodological framework beyond the current scope. Future research could explore these additional factors as complementary mechanisms, providing a more comprehensive understanding of the complex interplay that influences relationship satisfaction in couples coping with chronic illnesses such as PCa.

### Clinical implications

The findings of this study have several important clinical implications, particularly for the design of interventions aimed at supporting couples coping with PCa. First, the significant serial mediation of psychological flexibility and self-esteem bridging the relationship between PCa distress and relationship satisfaction suggests that targeting psychological flexibility could be the first key step for psychological interventions. This approach is particularly important because direct attempts to reduce distress alone are unlikely to have a significant effect on relationship satisfaction. Instead, enhancing psychological flexibility may enable individuals to better manage distress and support adaptive coping, in turn fostering higher self-esteem and improving relationship satisfaction. Therefore, therapeutic approaches such as acceptance and commitment therapy (ACT; [39]), which explicitly aim to increase psychological flexibility, may help couples to better cope with the emotional and psychological challenges of cancer. In addition, the finding of both distinguishability and interdependence between patients and partners may provide insight into the design of intervention formats. Distinguishability suggests that patients and partners may face unique challenges posed by PCa. Therefore, individualised interventions for both patients and partners may address their unique needs and maximise effectiveness. Interdependence also suggests that improvements in one partner may benefit the other. Thus, for both patients and partners, receiving individualised interventions may benefit not only themselves but also their partners. In particular, a recent systematic review of the effectiveness of psychological interventions for improving relationship satisfaction in couples coping with PCa found that individually delivered psychosocial interventions may be more effective than conjoint interventions, which may be due in part to challenges associated with the conjoint format, such as high dropout rates and ceiling effects [59]. Given these factors, providing psychological support in an individual format may be more practical for supportive care teams, as it may facilitate easier recruitment of representative samples and reduce dropout rates. Finally, the finding that partners tend to experience higher levels of distress, lower psychosocial flexibility, and lower self-esteem highlights the need for more interventions specifically tailored to partners, especially those in caregiving roles. Therefore, healthcare professionals should pay more attention to partners of cancer patients and provide them with the support and resources they need to cope with the emotional and practical challenges of caregiving.

## Conclusion

While it is well established that cancer distress can negatively impact individual well-being [75, 113], less is known about how it may affect relationship functioning beyond direct effects. In particular, while self-esteem has been well explored in PCa patients [6, 91], its role in a dyadic context, considering both patients and partners, has received little attention. Similarly, although psychological flexibility is well recognised as an adaptive strategy in individual mental health [23, 64], its crucial role in dyadic adjustment, particularly for couples coping with chronic illness, also needs to be better understood. Therefore, this study is the first to use the VSAM to understand the complex interplay between cancer-related distress, psychological flexibility, self-esteem, and relationship satisfaction in couples coping with PCa at both the individual and dyadic levels. By identifying both actor and partner effects, our findings highlight the distinguishability and interdependence between patients and partners in their experiences of how PCa distress affects relationship satisfaction. The findings underscore the importance of addressing dynamic traits, such as psychological flexibility, which may be the key step for interventions designed to support couples coping with cancer. These findings provide valuable guidance for clinicians in designing interventions that not only support patients but also address the unique emotional and psychological needs of partners. Finally, incorporating psychosocial support into multidisciplinary care teams (e.g. oncologists, psychologists, and social workers) can help provide more comprehensive care by including psychological assessments and tailored interventions that address the needs of both patients and their partners.

## Supplementary Information

Below is the link to the electronic supplementary material.

ESM 1 (DOCX 15.1 KB)

ESM 2 (DOCX 14.9 KB)

ESM 3 (DOCX 18.3 KB)

ESM 4 (DOCX 15.3 KB)

ESM 5 (DOCX 15.1 KB)

## Data Availability

Data cannot be shared openly but are available from the authors upon reasonable request.
