# Supplementary material for: Exploring the role of psychological flexibility in relationship functioning among couples coping with prostate cancer: a cross-sectional study
Source: Support Care Cancer. 2025 Feb 13;33(3):186. doi: 10.1007/s00520-025-09229-8 (PMC11821681; doi:10.1007/s00520-025-09229-8)
Supplement: Supplementary file 1 — (DOCX 15.1 KB) [file 520_2025_9229_MOESM1_ESM.docx]

**Supplementary Table 1** Missing data and Little’s MCAR results

| Variable | Missing Values (%) | |  |
| --- | --- | --- | --- |
|  | Patients | Partners |  |
| Age | 0.32 | 0.64 |  |
| TSD | 2.87 | - |  |
| PCD | 0.12 | 0.07 |  |
| RL | 0.32 | 0.64 |  |
| PF | 0.12 | 0.04 |  |
| RS | 0.09 | 0.09 |  |
| SE | 0.13 | 0.06 |  |
| Little’s MCAR | Chi-square | Degree of freedom | *p*-value |
|  | 4548 | 4474 | 0.218 |

MCAR = missing completely at random, PCD = prostate cancer distress, PF = psychological flexibility, SE = self-esteem, RL = relationship length, RS = relationship satisfaction, TSD = time since diagnosis. *Note*. Significant results are in bold (p < 0.05)
