# Supplementary material for: Exploring the role of psychological flexibility in relationship functioning among couples coping with prostate cancer: a cross-sectional study
Source: Support Care Cancer. 2025 Feb 13;33(3):186. doi: 10.1007/s00520-025-09229-8 (PMC11821681; doi:10.1007/s00520-025-09229-8)
Supplement: Supplementary file 2 — (DOCX 14.9 KB) [file 520_2025_9229_MOESM2_ESM.docx]

**Supplementary Table 2** Normality test results

| Variable | Patients | |  | Partners |  |  |
| --- | --- | --- | --- | --- | --- | --- |
|  | Skew | Kurtosis | K-S test (*p*) | Skew | Kurtosis | K-S test (*p*) |
| PCD | 0.53 | -0.45 | 0.050 | 0.39 | -0.64 | 0.081 |
| PF | 0.15 | -0.72 | 0.131 | 0.18 | -0.26 | 0.189 |
| SE | -0.51 | -0.16 | 0.041 | -0.21 | 0.04 | 0.201 |
| RS | -1.60 | 2.34 | < 0.01 | -1.14 | 0.74 | < 0.01 |

K-S = Kolmogorov-Smirnov, PCD = prostate cancer distress, PF = psychological flexibility, SE = self-esteem, RS = relationship satisfaction.
