# Supplementary material for: Exploring the role of psychological flexibility in relationship functioning among couples coping with prostate cancer: a cross-sectional study
Source: Support Care Cancer. 2025 Feb 13;33(3):186. doi: 10.1007/s00520-025-09229-8 (PMC11821681; doi:10.1007/s00520-025-09229-8)
Supplement: Supplementary file 3 — (DOCX 18.3 KB) [file 520_2025_9229_MOESM3_ESM.docx]

**Supplementary Table 3** Correlations matrix of continuous variables

| Variables | 1 | 2 | 3 | 4 | 5 | 6 | 7 | 8 | 9 | 10 | 11 | 12 |
| --- | --- | --- | --- | --- | --- | --- | --- | --- | --- | --- | --- | --- |
| 1. RS_T |  |  |  |  |  |  |  |  |  |  |  |  |
| 1. PCD_T | **-0.31** |  |  |  |  |  |  |  |  |  |  |  |
| 1. SE_T | **0.42** | **-0.59** |  |  |  |  |  |  |  |  |  |  |
| 1. PF_T | **0.36** | **-0.69** | **0.77** |  |  |  |  |  |  |  |  |  |
| 1. RS_R | **0.38** | **-0.13** | **0.22** | **0.13** |  |  |  |  |  |  |  |  |
| 1. PCD_R | -0.08 | 0.02 | -0.06 | 0.02 | **-0.16** |  |  |  |  |  |  |  |
| 1. SE_R | **0.12** | -0.01 | 0.03 | 0.00 | **0.30** | **-0.50** |  |  |  |  |  |  |
| 1. PF_R | **0.14** | -0.02 | 0.03 | 0.01 | **0.25** | **-0.63** | **0.72** |  |  |  |  |  |
| 1. Age_T | 0.03 | -0.09 | 0.02 | 0.04 | -0.02 | -0.08 | 0.07 | 0.05 |  |  |  |  |
| 1. RL | -0.08 | -0.07 | -0.02 | 0.04 | -0.05 | -0.05 | 0.06 | 0.05 | **0.47** |  |  |  |
| 1. TSD_T | 0.01 | -0.02 | 0.07 | 0.08 | -0.03 | 0.05 | 0.07 | 0.02 | **0.17** | **0.11** |  |  |
| 1. Age_R | 0.00 | -0.09 | 0.03 | 0.06 | 0.00 | -0.04 | 0.02 | 0.01 | **0.55** | **0.53** | 0.04 |  |

PCD = prostate cancer distress, PF = psychological flexibility, SE = self-esteem, R = partner, RL = relationship length, RS = relationship satisfaction, T = patient, TSD = time since diagnosis. *Note*. Significant results are in bold (p < 0.05)
