# Supplementary material for: Exploring the role of psychological flexibility in relationship functioning among couples coping with prostate cancer: a cross-sectional study
Source: Support Care Cancer. 2025 Feb 13;33(3):186. doi: 10.1007/s00520-025-09229-8 (PMC11821681; doi:10.1007/s00520-025-09229-8)
Supplement: Supplementary file 4 — (DOCX 15.3 KB) [file 520_2025_9229_MOESM4_ESM.docx]

**Supplementary Table 4** ANOVA test results of categorical variables

| Variable | Df | Sum of Squares | | F value | *p*-value |
| --- | --- | --- | --- | --- | --- |
| Education_T | 6 | | 39 | 0.272 | 0.950 |
| Employment_T | 5 | | 116 | 0.977 | 0.432 |
| Country_T | 4 | | 11 | 0.119 | 0.976 |
| Marital_T | 1 | | 14 | 0.584 | 0.445 |
| Phase_T | 2 | | 123 | 2.623 | 0.074 |
| Treatment_T | 16 | | 349 | 0.916 | 0.551 |
| Psyctreatment_T | 1 | | 8 | 0.335 | 0.563 |
| Education_R | 6 | | 182 | 1.272 | 0.270 |
| Employment_R | 4 | | 119 | 1.25 | 0.290 |
| Country_R | 4 | | 93 | 0.971 | 0.423 |
| Marital_R | 1 | | 1 | 0.031 | 0.860 |
| Psyctreatment_R | 1 | | 18 | 0.751 | 0.387 |

Df = degree of freedom, T = patient, R = partner.
