# Supplementary material for: Exploring the role of psychological flexibility in relationship functioning among couples coping with prostate cancer: a cross-sectional study
Source: Support Care Cancer. 2025 Feb 13;33(3):186. doi: 10.1007/s00520-025-09229-8 (PMC11821681; doi:10.1007/s00520-025-09229-8)
Supplement: Supplementary file 5 — (DOCX 15.1 KB) [file 520_2025_9229_MOESM5_ESM.docx]

**Supplementary Table 5** Model Fits

| Model | Fit | |  |  |  |  |
| --- | --- | --- | --- | --- | --- | --- |
|  | ${}^{2}$ | df | *p* | CFI | RMSEA | SRMR |
| Full | - | 0 | - | 1.000 | 0.000 | 0.000 |
| Actor-only | 32.683 | 12 | 0.001 | 0.979 | 0.074 | 0.056 |
| Constrained | 24.058 | 12 | 0.020 | 0.988 | 0.057 | 0.069 |
| Parsimonious | 10.956 | 9 | 0.279 | 0.998 | 0.026 | 0.034 |

CFI = comparative fit index, df = degree of freedom, RMSEA = root mean square error of approximation, SRMR = standard root mean square residual
